# Trend Analysis of Deaths With Unintentional Poisoning and Years of Life Lost in the South of Iran: 2004-2019

**DOI:** 10.34172/jrhs.2023.123

**Published:** 2023-09-29

**Authors:** Habibollah Azarbakhsh, Fatemeh Jafari, Seyed Parsa Dehghani, Hamed Karami, Jafar Hassanzadeh, Alireza Mirahmadizadeh

**Affiliations:** ^1^Student Research Committee, Shiraz University of Medical Sciences, Shiraz, Iran; ^2^Department of Health Policy Research Center, Institute of Health, Shiraz University of Medical Sciences, Shiraz, Iran; ^3^Department of Epidemiology, Research Center for Health Sciences, Institute of Health, Shiraz University of Medical Sciences, Shiraz, Iran; ^4^Non-Communicable Diseases Research Center, Shiraz University of Medical Sciences, Shiraz, Iran

**Keywords:** Unintentional poisoning, Mortality rate, Years of life lost, Joinpoint regression, Iran

## Abstract

**Background:** This study was conducted to determine the mortality rate and the years of life lost (YLL) due to unintentional poisoning in Fars province in the south of Iran.

**Study Design:** A cross-sectional study.

**Methods:** In this study, data from all of the deaths due to unintentional poisoning in the south of Iran between 2004 and 2019 was extracted from the population-based Electronic Death Registry System (EDRS). The Joinpoint Regression method was used to examine the trend of the crude mortality rate, the age-standardized mortality rate (ASMR), and the YLL rate.

**Results:** During the 16-year study period (2004-2019), 1466 deaths due to poisoning occurred in Fars province. Of this number, 75.2% (1103 cases) were in men, and 37.5% (550 cases) were in the age group of 15-29 years. The total YLL due to poisoning during the 16-year study period were 25149 and 8392 in men and women, respectively. According to the joinpoint regression analysis, the 16-year trend of YLL rate due to premature mortality was stable. Moreover, the annual percent change (APC) was -0.7% (95% CI: -4.0 to 2.7, *P*=0.677) for males and - 0.3% (95% CI: -3.8 to 3.3, *P*=0.862) for females.

**Conclusion:** The trend of crude mortality rate, ASMR and YLL due to unintentional poisonings was stable. Considering the high rate of mortality and YLL due to unintentional poisoning in the age group of 15-29 years, it is essential to take necessary actions in this age group.

## Background

 Poisoning is one of the most common injuries and the most common causes of hospitalization in emergency hospitals.^1^ The increase in the rapid process of industrialization and, subsequently, the increase in the number and types of chemicals have led to a greater prevalence of unintentional poisoning in different societies.^2^ In low- and middle-income countries, these poisonings can be much more serious following the consumption of kerosene, herbal medicines, insecticides, or herbicides.^3^ It affects all races and all ages and remains a leading cause of unintentional injury which may lead to permanent disability requiring long-term medical care.^2^ In 2020, adolescent deaths from unintentional drug overdoses surpassed deaths from cancer, among other leading causes of death.^4^ In 2015, the total cost of drug poisoning deaths among adolescents and young adults was estimated at $35.1 billion United States.^5^ In 2015, unintentional poisonings caused 86,400 deaths worldwide, with a mortality rate of 1.2 per 100 000.^6^ In an analysis of vital statistics in Canada (except one province) conducted from 2001 to 2007, unintentional poisonings were one of the three leading causes of death from unintentional injuries, and the rate of these poisonings was higher in men than in women.^7^ From 1999 to 2016 in the United States, changes in unintentional poisoning death rates showed increases for all racial/ethnic and age subgroups, most notably among whites in the 20-34 age group, and it also had a large increase in women, although it was less than men.^8^ In an epidemiological study related to unintentional poisoning with carbon monoxide (CO) gas in northwest Iran, the proportion of unintentional poisoning related to CO in relation to all poisonings was 11.6%.^9^

 Unintentional poisonings are significantly preventable and are important for public health. Years of life lost (YLL) is an epidemiological estimate of premature death. However, the burden caused by unintentional poisoning in low-income countries and different regions has not been well investigated.^10^ Therefore, various studies are vital for planning, preventive interventions, and optimizing the distribution of available resources. This study was conducted to determine the mortality rate and the YLL from unintentional poisoning in Fars province in the south of Iran.

## Methods

 This cross-sectional study was conducted in Fars province during 2004-2019. We extracted all deaths from unintentional poisonings from the population-based Electronic Death Registry System (EDRS) by age, gender, and year of death and based on International Classification of Diseases (ICD)-10. The codes used in this study were X40-X49. In the population-based electronic death registration system, all available sources were used to detect, record, and collect information about death, and then deaths that were repeated in records were excluded from the study. Inclusion criteria included death due to unintentional poisoning and being a resident of Fars province.

 The total estimated population of Fars province has been estimated using the basic data of health centers and the population and housing census from 1996 to 2016, taking into account the annual growth of the population. For standardization, the standard population of 2013 for countries with low and moderate incomes was used.^11^

###  Statistical analysis

 First, crude and age-standardized mortality rates (ASMR) of unintentional poisonings were calculated according to gender and year of death during the study years. Then, to calculate YLL, the standard life table was used, life expectancy for different age and gender groups was determined, and the number of deaths due to unintentional poisoning in each age and gender group was estimated based on the following relationship calculation^12^:

*YLL = N Ce^(ra)^/ (β + r)^2^ [e ^-(β + r)(L + a)^[-(β + r) (L + a)-1] – e^–(β + r)a^ [–(β + r) a-1]]*

 N = Number of deaths in a specified age and gender group

 L = Life expectancy of death cases again in that age and gender group

 r = Discounting Rate, which equals 0.03.

 β = A conventional rate in calculating age value which equals 0.04.

 C = An adjusted constant value that equals 0.1658.

 a = The age on which death occurred

 e = A constant value considered as 2.71.

 First, the YLL were calculated according to 18 age groups: 0-4, 5-9, 10-14, up to 85 years old, and then based on age groups 0-4, 5-14, 15-29, 30-44, 45-59, 60-69, 70-79, and over 80 years were shown in a figure.

 The analysis of the number of YLL due to premature death from unintentional poisonings was performed using the YLL template of 2015 (https://apps.who.int/gho/data/view.searo.60760?lang=en), World Health Organization (WHO) in Excel version 2016 spreadsheet software.

 To examine the trend of crude, ASMR, and YLL rate for different years, joinpoint regression based on the log-linear model was used. The joinpoint regression analysis describes changing trends over successive segments of time and the amount of increase or decrease within each segment. The resulting line segment between joint points is described by the annual percent change (APC) that is based on the slope of the line segment and the average annual percent change (AAPC). The joinpoint regression program 4.9.1.0 carried out the analysis for the trend, and *P* values < 0.05 were considered statistically significant. The protocol of this study was reviewed and approved by the Ethics Committee of Shiraz University of Medical Sciences. All aspects of the study were conducted according to the university’s code of ethics.

## Results

**Figure 1:**
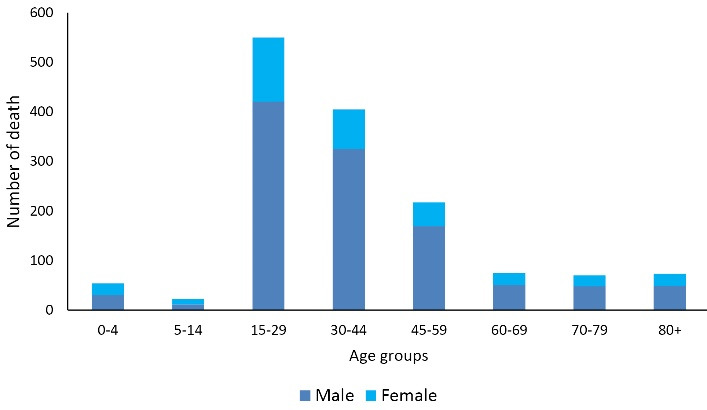
Number of deaths due to unintentional poisoning by gender and age groups

 During the 16-year study period (2004-2019), 1466 deaths due to poisoning occurred in Fars province. Of this number, 75.2% (1103 cases) were in men, and 37.5% (550 cases) were in the age group of 15-29 years. As can be seen in Table 1, the crude mortality rate due to poisoning in men increased from 4.73 (per 100 000 population) in 2004 to 5.84 per 100 000 population in 2019 (*P *for trend = 0.593, AAPC = -0.8%) and in women from 0.90 (per 100 000 population) in 2004 to 1.07 (per 100 000 population) in 2019. Furthermore, the ASMR had a stable trend in men from 4.73 per 100 000 in 2004 to 4.73 per 100 000 in 2019 (AAPC = -1.4%, *P* for trend = 0.349), and it increased from 0.83 per 100 000 population in 2004 to 0.96 per 100 000 in 2019 (AAPC = 0.8%, *P *for trend = 0.635) in women (Table 1). Moreover, the highest and lowest number of deaths in both genders were in the age groups of 15-29 years and 5-14 years, respectively (Figure 1).

**Table 1:** Crude and ASMR per 100 000 population and YLL due to poisoning by gender and year in Fars province during 2004-2019

**Year**	**No. of Death**		**Crude Mortality Rate**		**ASMR (95% CI)**		**YLL**			
							**No.**		**(Per 1000)**	
	**Male**	**Female**	**Male**	**Female**	**Male**	**Female**	**Male**	**Female**	**Male**	**Female**
2004	80	16	4.73	0.90	4.73 (3.79,5.67)	0.83 (0.39,1.27)	1749	427	0.94	0.24
2005	57	16	3.07	0.89	2.90 (2.10,3.70)	0.88 (0.44,1.32)	1351	385	0.72	0.21
2006	65	18	3.51	0.99	3.30 (2.44,4.15)	0.93 (0.47,1.39)	1520	456	0.82	0.25
2007	63	21	3.37	1.14	3.12 (2.29,3.95)	0.99 (0.50,1.48)	1435	507	0.76	0.27
2008	83	32	4.39	1.72	3.77 (2.82,4.72)	1.64 (1.04,2.23)	1977	790	1.04	0.42
2009	48	19	2.51	1.01	2.28 (1.56,2.99)	0.95 (0.50,1.41)	1014	383	0.53	0.20
2010	72	20	3.74	1.05	3.12 (2.26,3.99)	0.98 (0.51,1.44)	1680	517	0.87	0.27
2011	57	23	2.93	1.19	2.68 (1.92,3.44)	1.28 (0.79,1.77)	1227	560	0.63	0.29
2012	77	39	3.91	2.00	3.51 (2.64,4.39)	1.81 (1.18,2.44)	1667	803	0.84	0.41
2013	54	13	2.70	0.66	2.44 (1.72,3.16)	0.59 (0.23,0.95)	1288	267	0.64	0.13
2014	97	33	4.79	1.66	4.36 (3.40,5.31)	1.47 (0.90,2.03)	2290	748	1.13	0.37
2015	71	22	3.46	1.09	3.01 (2.20,3.81)	1.00 (0.54,1.45)	1691	493	0.82	0.24
2016	55	23	2.64	1.13	2.39 (0.61,1.54)	1.07 (0.61,1.54)	1210	489	0.58	0.24
2017	54	27	2.59	1.33	2.40 (1.70,3.09)	1.34 (0.84,1.85)	1218	679	0.58	0.33
2018	47	19	2.24	0.93	2.03 (1.39,2.68)	0.93 (0.51,1.36)	1029	438	0.49	0.21
2019	123	22	5.84	1.07	4.73 (3.70,5.76)	0.96 (0.51,1.41)	2803	450	1.33	0.22
Total	1103	363	3.50	1.17	3.13 (2.93,3.34)	1.12 (1.00,1.24)	25149	8392	0.79	0.27
*P*-value	-	-	0.593	0.658	0.349	0.635	-	-	0.677	0.862

*Note.* ASMR: Age-standardized mortality rate; YLL: Years of life lost; No: Number; CI: Confidence interval.

###  Temporal trends of poisoning mortality by age groups

 In the 0–44 age group, the poisoning mortality rate had decreasing trends in men (AAPC = –0.4%, *P* = 0.833) and women (AAPC = –1.2%, *P* = 0.511), but these trends were not statistically significant. In the 45–59 age group, there were increasing trends in men (AAPC = 1.9%, *P* = 0.384) and women (AAPC = 4.7%, P = 0.019), which were not statistically significant. Moreover, in the 60–74 age group, there were decreasing trends in men (AAPC = -6.01%, *P* = 0.091) and women (AAPC = -1.2%, *P* = 0.568), but these trends were not statistically significant. Likewise, in the + 75 age group, there were decreasing trends in men (AAPC = -6.3%, *P* = 0.081) and women (AAPC = -4.4%, *P* = 0.327), but these trends were not statistically significant.

###  Temporal trends of poisoning years of life lost rate 

**Figure 2:**
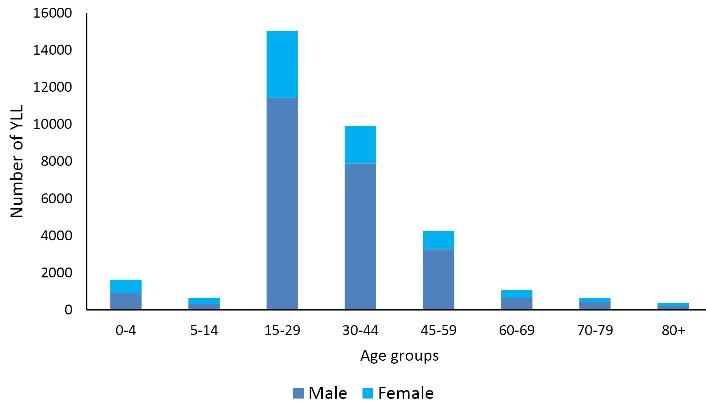
YLL due to unintentional poisoning by gender and age groups. *Note.* YLL: Years of life lost

 As depicted in Table 1, the total YLL due to poisoning during the 16-year study period was 25149 (0.79 per 1000 people) in men, 8392 (0.27 per 1000 people) in women, and 33541 (53.0 per 1000 people) in both genders (Male/female gender ratio = 2.99). The average number of YLLs due to unintentional poisoning was 22.8 years in men and 23.1 years in women, and the highest and lowest YLLs in both genders were in the age groups of 15-29 years and above 80 years, respectively, as illustrated in Figure 2.

**Figure 3:**
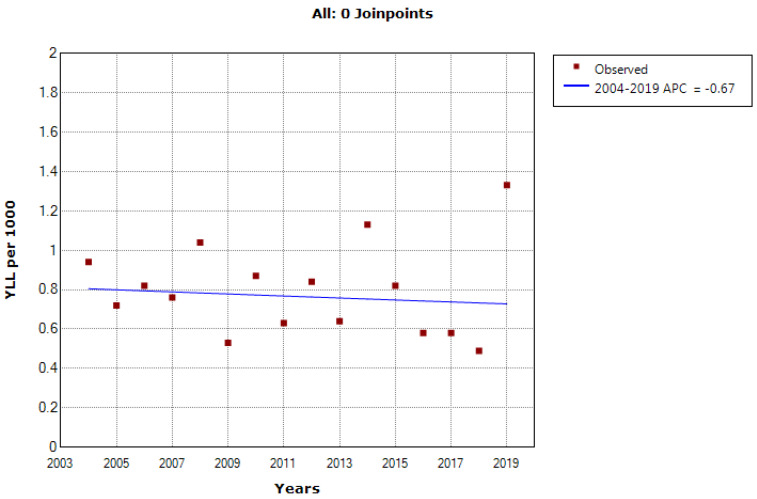
The trend of the number of YLL due to unintentional poisoning in men during 2004-2019. *Note.* YLL: Years of life lost

**Figure 4:**
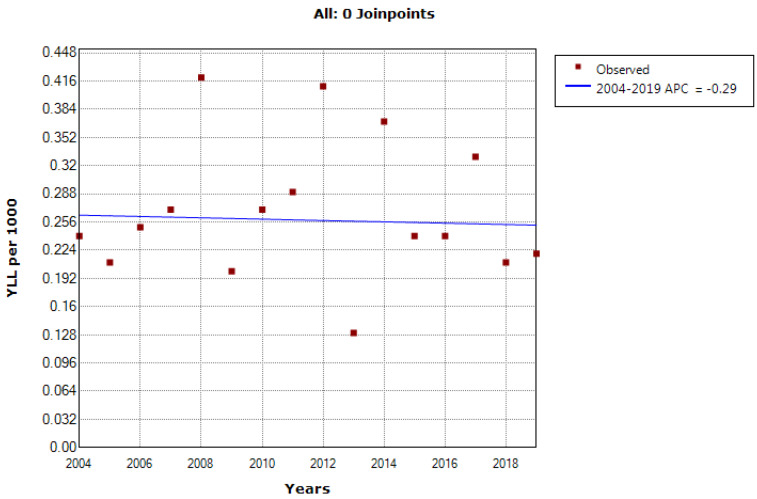
The trend of the number of YLL due to unintentional poisoning in women during 2004-2019. *Note.* YLL: Years of life lost.

 According to the joinpoint regression analysis, the 16-year trend of the YLL rate due to premature mortality was stable. The APC was -0.7% (95% confidence interval [CI]: -4.0 to 2.7, *P* = 0.677) for males and -0.3% (95% CI: -3.8 to 3.3* P* = 0.862) for females. The model did not show any joinpoint; hence, the AAPC was the same as the APC (Figures 3 and 4).

## Discussion

 The present study was conducted to investigate the mortality rate and YLL caused by unintentional poisoning in Fars province during 2004-2019. During the 16-year study period, 1,466 deaths due to poisoning occurred in Fars province, and the total YLL was estimated to be 25 149. In other studies, these numbers were reported to be higher. For example, in the United States, a total of 21 689 youth (aged 10–24) died of unintentional drug overdoses, and youth experienced a total of 1 227 223.58 YLLs during the 5-year study period (2015–2019).^13^ In China. the number of unintentional poisoning deaths decreased from 43,601 (in 1990) to 22,274 (in 2015), and the death rate decreased from 4.1 per 100 000 to 1.6 per 100 000 which indicates a decrease of 61.8%,^2^ while in our study, the trend of crude mortality in both genders was increasing. In the evaluation of patients who died from aluminum phosphide (rice tablets) in two years in Iran, the trend of crude mortality showed an increasing trend.^14^ Additionally, Hempstead and Phillips reported that the mortality rate from unintentional drug poisoning also increased by approximately 126% from 2005 to 2016.^15^ The high rate of mortality due to poisoning can have various causes such as improper use of medicine,^16^ use of pesticides,^17^ changes in the availability of drugs,^18^ access to medical centers, weak pre-hospital care, and the like,^19^ so there is an important point that poisoning requires a disinfection method to be done as soon as possible.^20^

 One study reported that the ASMR of death from acute pesticide poisoning decreased from 5.8 per 100 000 in 2006 to 1.6 per 100 000 in 2014 which was greater in men,^21^ but in our study, this measurement was stable in men and increased in women. There may be several reasons for the declining trend of mortality. For example, better access to health care services may have occurred due to increased urbanization^22^ or weak to non-existent surveillance system for poisoning exposures and inappropriate definition of poisoning cause undercounting.^23^ Another study demonstrated that men have more ASMR of death due to unintentional poisonings than women.^15^ In line with our study, Onyeka demonstrated a higher YLL rate in men compared to women.^24^ Higher mortality and consequently more YLL in men could be because this group has more access to toxic substances.^25^ Other research has suggested that the higher death rate in men than in women may be due to their greater involvement in risky behaviors such as working with tools, burning fuel, or consuming alcohol or working more at risky conditions.^9^

 Unlike our study, YLL due to poisoning increased during the 16-year study period in both genders, but in Tang’s study, there was a downward trend until 2015.^2^ A study conducted in Mexico on YLL of alcohol revealed that YLL in men and women peak in the age groups of 45-49 years and 50-54 years, respectively.^26^ Besides, in a study conducted in Iran, the results illustrated that during 10 years (2004-2013), the age group of 15-29 had the highest amount of YLL in both genders,^27^ and the present study also obtained similar results in this regard. It may indicate increased mortality in future years for all ages,^4^ and the highest burden of disease will be on the shoulders of the youngest and most active people in society; hence, taking measures to reduce or better manage patients will have a great impact on social welfare.^27^ On the other hand, this age group is considered to be an active part of the society, and they are facing more dangers due to their greater presence in society and higher risk-taking.

 The findings of this study have important implications for policymaking and the development of appropriate poisoning interventions. If resources are limited, YLL may particularly help policymakers target premature death subpopulations and set top priorities for programs related to the prevention of premature death.^28^ For example, uninformed and incorrect social attitudes toward the recreational use of prescription opioids may be a reason for the widespread use of drugs. Furthermore, despite the common belief that poisoning is solely the result of heavy alcohol or drug use, the findings of Yoon and colleagues’ study suggested that poisoning is more likely due to chronic substance use disorders and mood and anxiety disorders.^29^ Greater symptom severity or the availability of psychiatric medications can lead to higher risks for accidental poisoning in people with a major mental health disorder.^2^ Therefore, it is suggested that poison control centers with telephonic advice services should be readily available to handle poisoning cases with confidence,^19^ or people should be provided with sufficient training regarding the correct way and amount of using medicine or chemicals.^30^ Additionally, it is important to know that research and policy attention to poisoning has been relatively low compared to the magnitude of the problem, especially among young people, so further studies are recommended.

 A limitation of the present study was that YLL was not evaluated throughout Iran due to the unavailability of the necessary data. In addition, the data used in this study were not sufficient to determine the specific risk factors of unintentional poisoning in South Asian countries. However, this study has a large sample size and an extensive time period for data analysis.

**Highlights**

- During the 16 years of study (2004-2019), 1466 deaths occurred in Fars province due to Unintentional poisoning.
- The total YLL due to poisoning during the 16-year study period were 25,149 in men and 8392 in women.
- According to the joinpoint regression analysis, the 16-year trend of YLL rate due to premature mortality was stable in both genders.

## Conclusion

 During the study period, the trend of crude and standardized mortality rates and YLL due to unintentional poisonings has been stable. Considering the high rate of mortality and YLL due to unintentional poisoning in the age group of 15-29 years, it is essential to take necessary actions in this age group.

## Acknowledgments

 We would like to acknowledge the Health Vice-chancellor at Shiraz University of Medical Sciences.

## Authors’ Contribution

**Conceptualization:** Habibollah Azarbakhsh, Alireza Mirahmadizadeh.

**Data curation:** Fatemeh Jafari, Jafar Hassanzadeh.

**Formal analysis:** Habibollah Azarbakhsh.

**Funding acquisition:** Alireza Mirahmadizadeh.

**Investigation:** Alireza Mirahmadizadeh.

**Methodology:** Habibollah Azarbakhsh.

**Project administration:** Jafar Hassanzadeh, Seyed Parsa Dehghani.

**Software:** Habibollah Azarbakhsh.

**Supervision:** Habibollah Azarbakhsh.

**Validation:** Habibollah Azarbakhsh, Fatemeh Jafari, Hamed Karami.

**Visualization:** Jafar Hassanzadeh, Seyed Parsa Dehghani.

**Writing–original draft:** Habibollah Azarbakhsh, Fatemeh Jafari, Hamed Karami, Seyed Parsa Dehghani.

**Writing–review & editing:** Habibollah Azarbakhsh, Fatemeh Jafari, Seyed Parsa Dehghani, Hamed Karami, Jafar Hassanzadeh, Alireza Mirahmadizadeh.

## Competing Interests

 The authors announce that they have no conflict of interests in the publication of this study. The Local Ethics Committee approval was also obtained.

## Funding

 None.
